# *ahg12* is a dominant proteasome mutant that affects multiple regulatory systems for germination of *Arabidopsis*

**DOI:** 10.1038/srep25351

**Published:** 2016-05-03

**Authors:** Shimpei Hayashi, Takashi Hirayama

**Affiliations:** 1 Graduate School of Nanobioscience, Yokohama City University, 1-7-29 Suehiro, Tsurumi, Yokohama 230-0045, Japan; 2 Institute of Plant Science and Resources, Okayama University, 2-20-1 Chuo, Kurashiki, Okayama 710-0046, Japan

## Abstract

The ubiquitin-proteasome system is fundamentally involved in myriad biological phenomena of eukaryotes. In plants, this regulated protein degradation system has a pivotal role in the cellular response mechanisms for both internal and external stimuli, such as plant hormones and environmental stresses. Information about substrate selection by the ubiquitination machinery has accumulated, but there is very little information about selectivity for substrates at the proteasome. Here, we report characterization of a novel abscisic acid (ABA)-hypersensitive mutant named *ABA hypersensitive germination12* (*ahg12*) in Arabidopsis. The *ahg12* mutant showed a unique pleiotropic phenotype, including hypersensitivity to ABA and ethylene, and hyposensitivity to light. Map-based cloning identified the *ahg12* mutation to cause an amino acid conversion in the L23 loop of RPT5a, which is predicted to form the pore structure of the 19S RP complex of the proteasome. Transient expression assays demonstrated that some plant-specific signaling components accumulated at higher levels in the *ahg12* mutant. These results suggest that the *ahg12* mutation led to changes in the substrate preference of the 26S proteasome. The discovery of the *ahg12* mutation thus will contribute to elucidate the characteristics of the regulated protein degradation system.

As sessile organisms, plants cannot move and therefore need internal mechanisms to respond to environmental conditions. Accordingly, plants have developed unique response and adaptation systems to cope with the environment, including environmental stresses. Physiological and genetic studies have shown that plants are able to sense tiny environmental changes, such as fluctuations in light intensity, CO_2_ concentration, temperature, various chemicals, minerals, and water pressure, as well as touch and attack by pathogens or animals. The stimuli received from environmental changes are transduced to nuclei, where these multiple stimuli seem to be processed and integrated to evoke adequate and timely regulation of sets of genes for environmental adaptation^1^,^2^,^3^,^4^,^5^,^6^.

Recent studies on gene regulatory systems in plants have highlighted the importance of the ubiquitin-proteasome protein degradation system in the modulation of gene expression responding to environmental and developmental stimuli^7^,^8^. Light is an important signal for photosynthetic organisms and represents one of the major developmental cues for plants, regulating germination, skotomorphogenesis, flowering, and senescence^1^. The activity of key transcriptional factors, such as HY5 and PIFs, in the gene regulatory system responsive to light stimuli, is modulated by the ubiquitin proteasome system^9^.

Plant hormones also play pivotal roles in developmental processes and responses to environmental stresses. These compounds function as signaling molecules in the long-distance cell-cell communication systems in plants^10^,^11^. In the last decades, the molecular mechanisms underlying the signaling pathways of plant hormones from signal perception to the resulting gene regulation have been elucidated significantly. Interestingly, proteasomal protein degradation is deeply involved in the all of the plant hormone signaling pathways^7^,^12^. For example, indole acetic acid (IAA), or auxin, which is major developmental regulator, is recognized by a protein complex composed of a ubiquitin E3-ligase named TIR1 and its target protein AUX/IAA^13^,^14^,^15^. IAA stabilizes the interaction between TIR1 and AUX/IAA and facilitates the degradation of this inhibitory protein to activate auxin-responsive gene expression. In addition, EIN3, the pivotal transcription factor in the ethylene signaling pathway, is unstable and degraded by the ubiquitin-proteasome system in the absence of ethylene, whereas it becomes stable and activates ethylene-responsive genes in the presence of ethylene^16^,^17^. The proteasome is also involved in responses to abscisic acid. ABI5 and ABI3, important transcription factors involved in the abscisic acid response in the early germination stage, are targets of the ubiquitin-proteasome system^18^,^19^.

The proteasome is very large protein complex composed of the catalytic 20S core particle (CP) and 19S regulatory particle (RP)^20^. The CP comprises four heptameric rings (two α1-α7 rings and two β1-β7 rings) forming a barrel-like structure, in which the substrate protein is degraded in an ATP-dependent manner. The RP consists of 19 subunits and can be subdivided into the lid subcomplex and the base subcomplex. The lid subdomain consists of non-ATPase subuinit proteins RPN1-RPN13. Among them, RPN10 and RPN13 have the ability to bind ubiquitin while RPN11 removes ubiquitins from the ubiquitinated proteins^21^,^22^,^23^, suggesting that the lid subcomplex is involved in the recognition of ubiquitinated proteins . The base subcomplex consists of six homologous AAA-ATPases, RPT1-RPT6. These ATPases are thought to unfold the traget proteins and deliver them to the CP^24^,^25^ . Structural analyses of the RP have revealed the relative position of each component and are consistent with the predicted functions of these proteins^26^,^27^,^28^.

Accumulated evidence suggests that each subunit of RP has specific functions in plant physiological phenomena, presumably through the regulation of protein degradation. RPN10- and RPN12-defective mutants are altered in the responses to plant hormones such as auxin and cytokonin in Arabidopsis^29^,^30^. Mutations in RPN1 of Arabidopsis cause embryogenesis and growth defects^31^,^32^. Studies of RPT2 mutants indicated RPT2 is required for meristem maintenance and gametophyte or sporophyte development^33^,^34^ and that RPT2 is involved in gene silencing via DNA methylation^35^. A loss-of-function mutation of *RPT5a*, one of the two *RPT5* genes in Arabidopsis, results also in defects in sporophyte development^36^. Furthermore, RPT2a and RPT5a are required for Zinc deficiency-tolerance in Arabidopsis^37^. This non-redundancy in the function of RPT1-6 has also been reported in the yeast system^25^,^38^.

Here we report the novel *RPT5a* allele of Arabidopsis, *ABA hypersensitive germination 12* (*ahg12*). The *ahg12* mutant was isolated based on its weak ABA hypersensitivity at the germination stage^39^. In this study, we found that this mutant has a pleiotropic phenotype, including ethylene hypersensitivity and diminished dormancy. We found that the *ahg12* mutation alters an amino acid residue in RPT5a. This amino acid residue is outside of the ATPase domain but is highly conserved among RPT5 orthologs. Recent structural analyses of the PAN complex of the archaea *Mathanocoldococcus jannaschii* and the RP complex of fission yeast revealed this residue to be in the L23 loop, which is predicted to form a pore structure of the base subcomplex^40^,^41^,^42^. Detailed analysis of the *ahg12* mutation will provide information related to the function of RPTs and the RP subcomplex.

## Results

### *ahg12* exhibits a pleiotropic phenotype in germination

**Figure 1:**
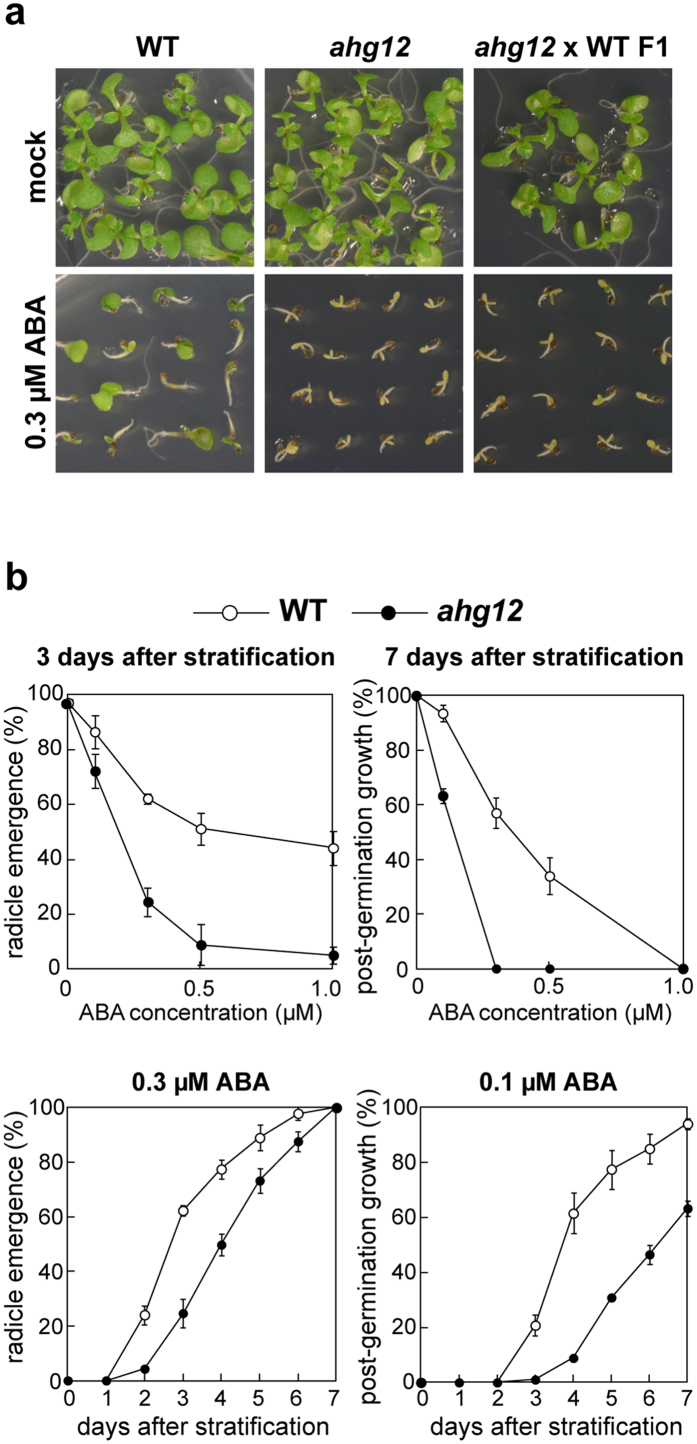
Seed germination of *ahg12* is hypersensitive to ABA. (**a**) Germinating seeds of *ahg12* in the presence of ABA. Imbibed and stratified seeds of wild type (WT), *ahg12*, and F1 progeny between *ahg12* and WT were grown on plates containing ABA for 7 days. (**b**) Germination rate of *ahg12* in the presence of ABA. Imbibed seeds (>50) of WT and *ahg12* were stratified and then sown on plates containing ABA. Seeds that showed radicle emergence or post-germination growth (expansion of green cotyledons) were counted. The data are mean of three independent experiments. Error bars indicate standard deviation.

Compared to the wild type, *ahg12* mutant shows greater growth inhibition in response to exogenously applied ABA during seed germination using radicle emergence and post-germination growth as phenotypic markers (Fig. 1). It is unlikely that the enhanced ABA response in the mutant is conferred by an increased amount of endogenous ABA, because the *ahg12* mutant was isolated originally from a mutagenized population of *aba2-1* seeds that are deficient in ABA biosynthesis and is not the reversion mutant of *aba2-1* regarding its mapped position^39^. Therefore, the *ahg12* mutant is more likely to have changes in sensitivity to ABA. The ABA-hypersensitive phenotype was also observed in the F1 progeny of a cross between *ahg12* and wild-type plants, indicating that the *ahg12* mutation confers ABA hypersensitivity in a dominant manner. To investigate the ABA sensitivity of *ahg12* at the seedling stage, we measured the root elongation rate. We found no significant differences between wild type and *ahg12* in root growth rate at the seedling stage in the presence of various concentrations of ABA (Fig. S1). These findings suggest that the ABA-hypersensitive phenotype of *ahg12* is restricted to the germination or early germination stage.

**Figure 2:**
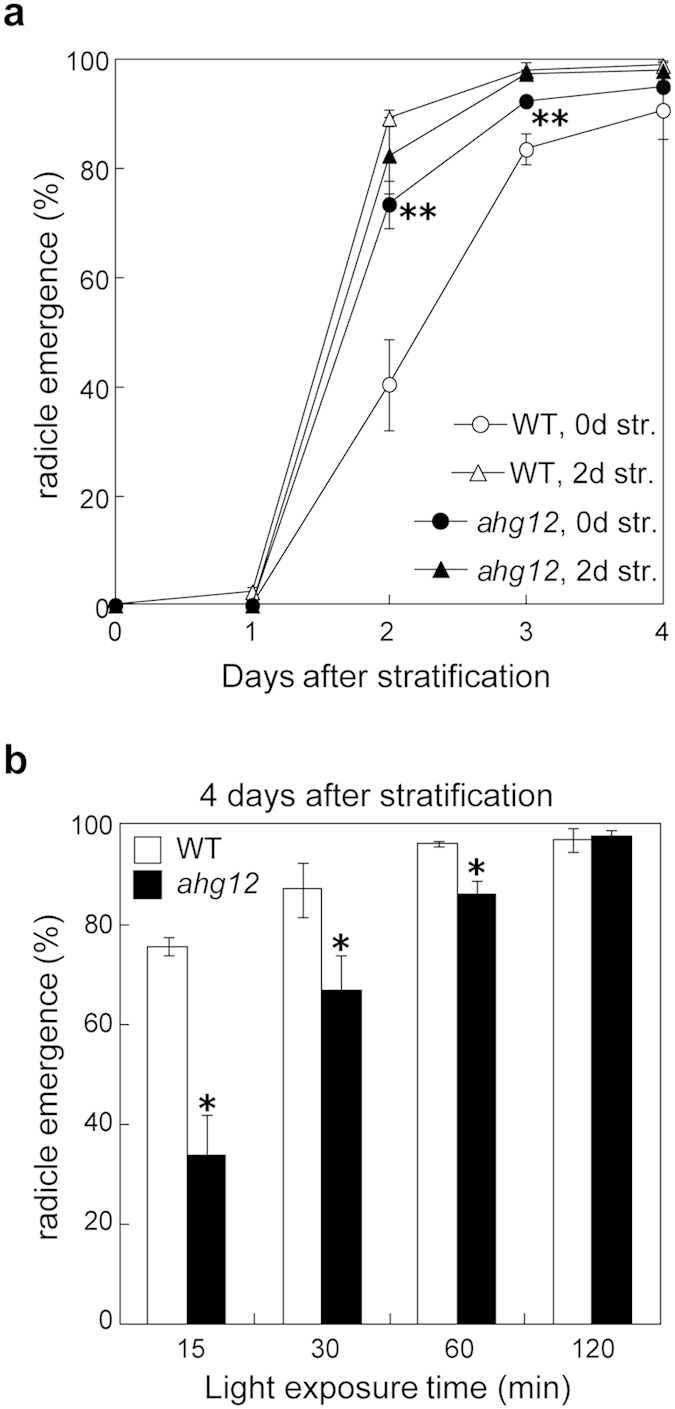
Pleiotropic phenotype of *ahg12.* (**a**) Efficiency of stratification in *ahg12*. Imbibed seeds (>60) of *ahg12* and WT were incubated at 4 °C in the dark for the indicated periods (0 or 2 days) and then grown under normal conditions. Seeds that showed radicle emergence were counted every day. The data are means of three independent experiments. Error bars indicate standard deviation. (**P < 0.01; t-test after arcsine-transformation) (**b**) Efficiency of light exposure to induce seed germination. Imbibed and stratified seeds of *ahg12* and WT were exposed to the light (white fluorescent light, about 150 E/s.m^2^) for the indicated periods (15, 30, 60, and 120 min). The seeds (>50) were grown in dark for 4 days, and then germinated seedlings were counted. The data are means of three independent experiments. Error bars indicate standard deviation. (*P < 0.05; t-test after arcsine-transformation).

ABA is involved in the seed dormancy. In some cases, a positive correlation between ABA sensitivity and seed dormancy has been observed^43^,^44^. To investigate seed dormancy of the *ahg12* mutant, we compared the effects of various stratification periods on seed germination between *ahg12* and wild type using seeds harvested on the same date. Interestingly, without stratification, the *ahg12* mutant showed higher germination rates than wild type (Fig. 2a). This result indicated that the *ahg12* mutant displayed lower seed dormancy despite its enhanced ABA sensitivity. After a 2-day stratification, the seed germination rate of *ahg12* was almost equal that of wild type.

The above results suggested that the physiological status of the *ahg12* seed during germination is markedly different from that of wild type. Thus, the responses of *ahg12* at the germination stages to other stimuli were also investigated. Light is an important stimulus that affects germination of Arabidopsis. Accordingly, we investigated the germination rates of stratified seeds exposed to light for different lengths of time. The *ahg12* seeds required longer light exposure to reach a similar rate of germination as the wild-type seeds (Fig. 2b), indicating a reduced responsiveness of *ahg12* to light during seed germination.

**Figure 3:**
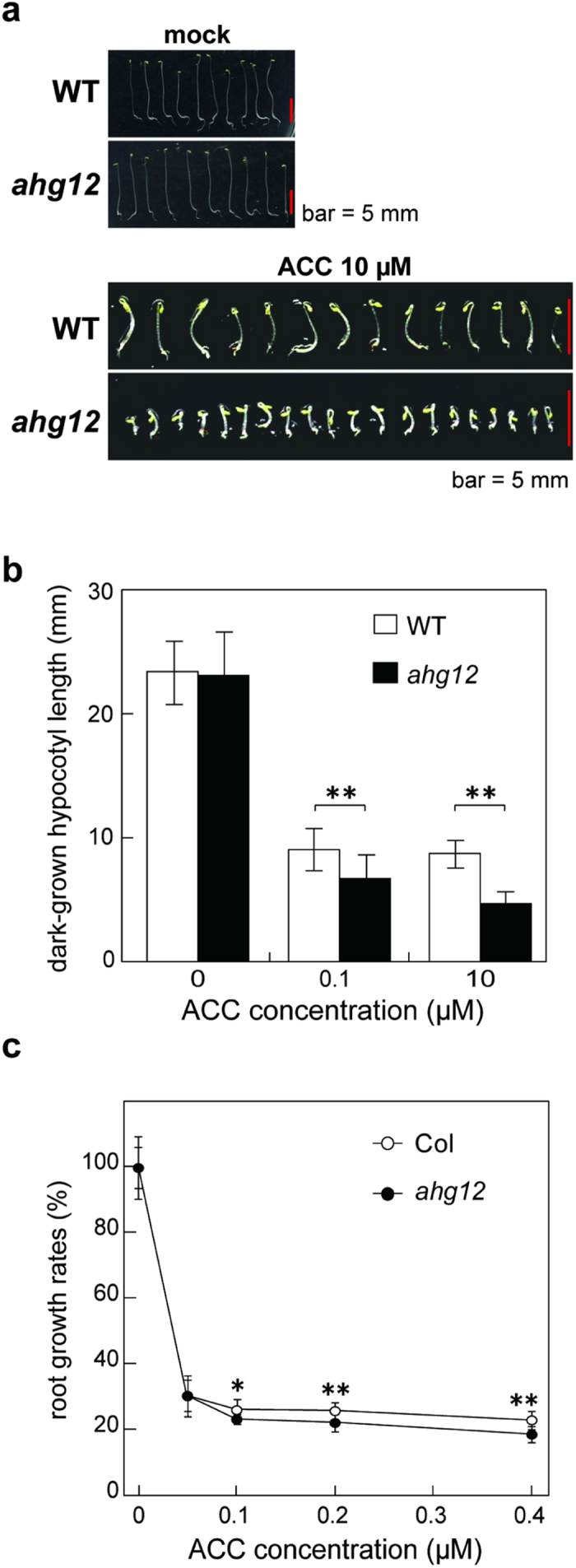
a*hg12* is hypersensitive to ethylene. (**a**) Etiolated seedlings of *ahg12* grown on plates containing ethylene precursor. Imbibed seeds of wild type (WT) and *ahg12* were stratified for 2 days and then grown on plates with or without 1*-*aminocyclopropane*-*1*-*carboxylic acid (ACC, 10 μM) for 4 days. (**b**) Hypocotyl length of the etiolated *ahg12* seedlings grown on plates containing ethylene precursor. Hypocotyl lengths of the etiolated seedlings (>20) grown on plates containing various concentrations (0, 0.1, and 10 μM) of ACC for 4 days were measured. (**c**) Root growth rate of *ahg12* seedlings grown on plates containing ethylene precursor. Root length of 4-day-old seedlings grown on normal plates were measured, and seedlings were transferred to ACC-containing plates. After 4 days, root length was measured again to calculate the growth rate. The data are means of three independent experiments. Error bars indicate standard deviation. Asterisks indicate significant differences between the corresponding values. (*P < 0.05; **P < 0.01; t-test after arcsine-transformation).

We next investigated responses of the *ahg12* mutants to other phytohormones. The growth-inhibition or -promotion effects of auxin, cytokinin, and gibberellin were not markedly different between *ahg12* and wild type in terms of hypocotyl length and root elongation rates (Fig. S2). Interestingly, there was a significant difference in sensitivity to ethylene. When grown on medium containing the ethylene precursor 1*-*aminocyclopropane*-*1*-*carboxylic acid (ACC), the *ahg12* mutant showed clearly shorter roots and dark-grown hypocotyls than wild type, indicating a hypersensitivity to ethylene in the mutant (Fig. 3).

Taken together, our results demonstrate that *ahg12* is a mutant with a unique combination of phenotypes: increased sensitivity to ABA, decreased dormancy, decreased responsiveness to light during seed germination, and increased sensitivity to ethylene.

### The *ahg12* mutation localizes to the pore structure of the RP complex

**Figure 4:**
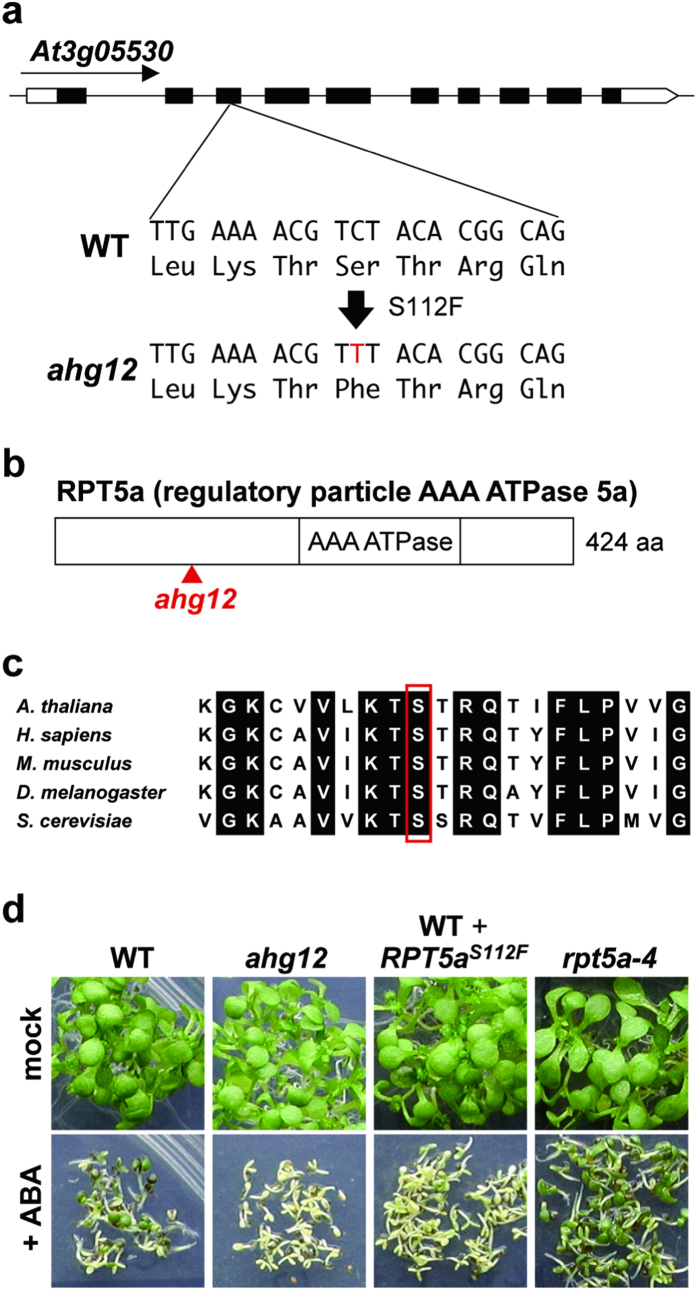
a*hg12* is a novel mutant allele of *RPT5a.* (**a**) Schematic representation of the *AHG12* gene and the *ahg12* mutation site. A transition mutation (C to T) was detected at codon 112 of the *At3g05530* gene in *ahg12*. White and black boxes indicate untranslated regions and exons, respectively. (**b**) Schematic representation of AHG12/RPT5a protein. The approximate position of the *ahg12* mutation site is shown. (**c**) Alignment of the polypeptide sequences around the amino acid residues corresponding to the *ahg12* mutation site (red square) of RPT5 from various organisms. Identical amino acid residues are shown with a black background. (**d**) ABA sensitivity of transgenic plants expressing a modified *RPT5a* gene and T-DNA disruptants of *RPT5a* (*rpt5a-4*). Genomic *RPT5a* with the *ahg12* mutation containing putative promoter and terminator regions was introduced into WT plants (WT + *RPT5a*^*S112F*^). Imbibed and stratified seeds were sown on plates with or without ABA (0.3 μM) and grown for 7 days.

To identify the *ahg12* mutation, genetic linkage analysis of the F2 generation (approximately 800 lines) of a test cross between *ahg12* (Col) and wild type (L*er*) was undertaken. The ABA-hypersensitive phenotype was linked to a genomic region containing 35 genes on chromosome 3. Using DNA sequencing analysis, a point mutation consistent with effect of the EMS mutagen used to generate these mutants was identified in the *At3g05530* gene, which encodes the proteasome RP AAA-ATPase 5a (RPT5a) (Fig. 4a,b). The mutation was located in the third exon of the *RPT5a* gene and caused an amino acid substitution, Ser112 to Phe. Amino acid sequences of RPT5 orthologs are conserved in eukaryotes such as yeasts and animals, and this Ser112 residue is exceptionally highly conserved (Fig. 4c). However, mutants altered at the corresponding amino acid residue have not been reported previously.

To confirm that this mutation is responsible for the ABA hypersensitivity, we generated the transgenic plants expressing the *RPT5a* gene with the identified mutation and investigated their ABA sensitivity in seed germination. In spite of possessing a wild-type copy of *RPT5a* as an endogenous gene, the transgenic plants expressing the *ahg12* allele showed higher sensitivity to ABA than wild type (Fig. 4d). This dominant effect of the transgene was consistent with the dominancy of *ahg12*, demonstrating that the identified mutation is responsible for the ABA hypersensitivity of *ahg12*. We also investigated the ABA sensitivity of a T-DNA insertional disruptant mutant of *RPT5a* (*rpt5a-4*, SALK_046321, Fig. S3) as a second allele of *ahg12*. However, the *rpt5a-4* mutant did not show ABA hypersensitivity (Fig. 4d). This difference between phenotypes suggests that *ahg12* does not cause inactivation of RPT5.

**Figure 5:**
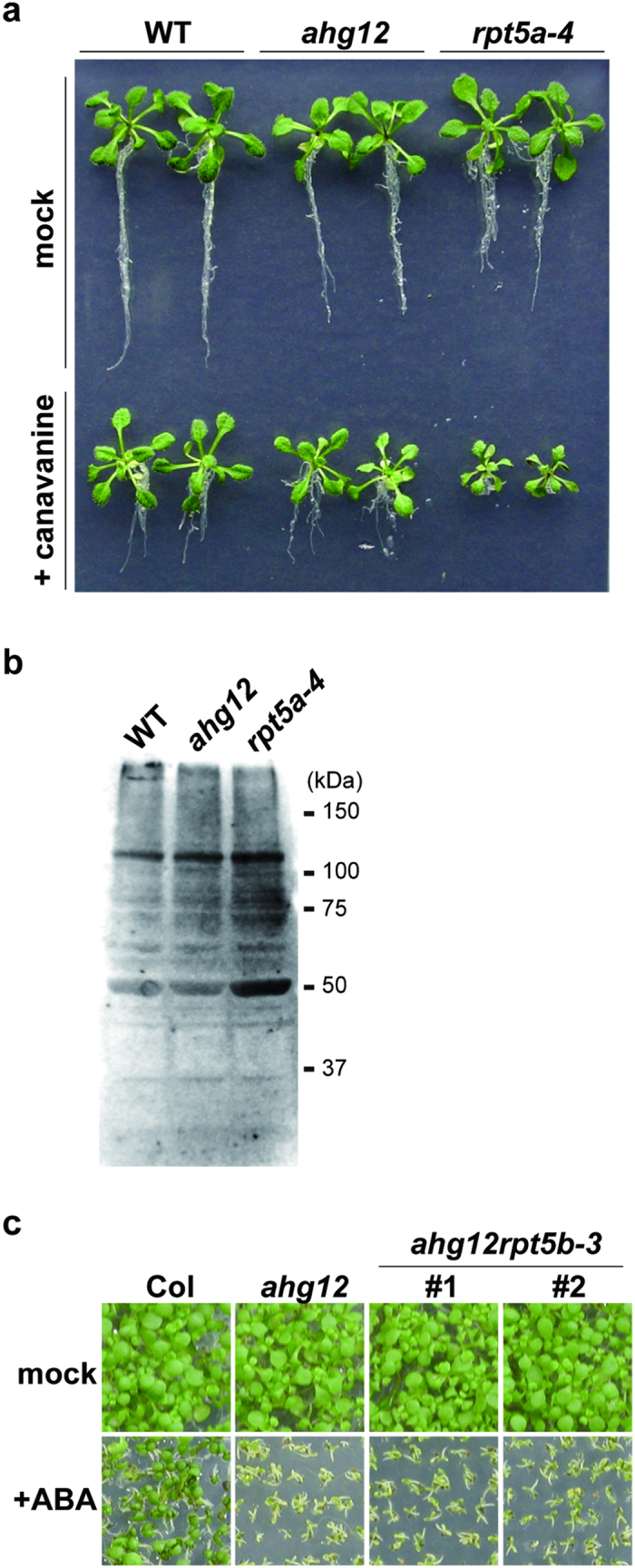
The 26S proteasome in *ahg12* remains essentially functional. (**a**) Canavanine sensitivity of *ahg12*. Imbibed and stratified seeds of WT, *ahg12*, and disruptant of RPT5a (*rpt5a-4*) were grown on plates containing 8 μM canavanine for 4 weeks. (**b**) Ubiquinated proteins in *ahg12* seedlings. Total proteins extracted from 2-week-old seedlings of WT, *ahg12*, and *rpt5a-4* were resolved by SDS-PAGE and ubiquitinated proteins were detected by immunoblot. (**c**) ABA sensitivity of the *ahg12* and *ahg12rpt5b-3* mutants at germination. Imbibed and stratified seeds of WT, *ahg12*, and *ahg12rpt5b-3* were sown on plates with or without ABA (0.3 μM) and grown for 7 days.

RPT5 is a subunit of the 26S proteasome complex. The dominant effect of *ahg12* implied that the mutation affected some function of this complex. To investigate whether the *ahg12* mutant is deficient in the protein degradation mediated by the proteasome, the mutant’s sensitivity to canavanine was evaluated. Canavanine, an analog of arginine, is incorporated into newly synthesized proteins and changes physicochemical properties of the proteins. Mutants that are deficient in the ubiquitin-proteasome system show increased sensitivity to the toxicity of proteins containing canavanine^32^,^45^. Consistent with previous studies, *rpt5a-4* showed higher sensitivity to canavanine than wild type, probably due to a lower capacity for removal of toxic proteins (Fig. 5a). This growth-inhibitory effect was prominently visible in the root tissues, which were directly in contact with the medium containing canavanine. By contrast, the canavanine sensitivity of *ahg12* was almost the same as that of wild type. This result suggests that the proteasome-mediated protein degradation system is essentially functional in the *ahg12* mutant. Consistent with this, the levels of total polyubiquitinated proteins determined by immunoblot analysis using with an antibody recognizing ubiquitinated proteins were similar between *ahg12* and wild type (Fig. 5b).

Arabidopsis has two genes that encode RPT5: *RPT5a* and *RPT5b* (Fig. S4). To investigate the possibility that the presence of *RPT5b* contributed to the ABA hypersensitivity in *ahg12*, we generated a double mutant between *ahg12* and a T-DNA disruption mutation in *RPT5b* (*rpt5b-3*). The double mutant showed ABA hypersensitivity during seed germination similar to that of the *ahg12* single mutant (Fig. 5c, Fig. S5), demonstrating that the ABA-hypersensitive phenotype in *ahg12* is independent of RPT5b. It was reported that dysfunction of both RPT5a and RPT5b leads to sterility^36^. However, we successfully obtained the double mutant between *ahg12* and *rpt5b*. This result supports the idea that the RPT5a^*ahg12*^ is functional as RPT5.

To examine potential functional differences between RPT5a and RPT5b, a recombinant *RPT5b* gene with an *ahg12*-like mutation (Ser111 to Phe, *RPT5b*^*S111F*^, Fig. S4) was introduced into wild-type plants. However, unlike the case of *ahg12*, the obtained transgenic plants, in which *RPT5b*^*S111F*^ is the major *RPT5b* transcript, did not show any ABA hypersensitivity during seed germination (Fig. S6). This result suggests that there are functional differences other than the gene expression pattern between *RPT5a* and *RPT5b*.

Since serine is the major target for protein phosphorylation in eukaryotes, we speculated that the Ser112 of RPT5a might be a phosphorylation site. To investigate this possibility, we generated a construct for recombinant RPT5a in which Ser112 was converted to aspartic acid to mimic phosphorylation and introduced it to wild-type plants. We obtained several independent transgenic lines but did not detect any abnormal ABA sensitivity in germination (Fig. S6).

**Figure 6:**
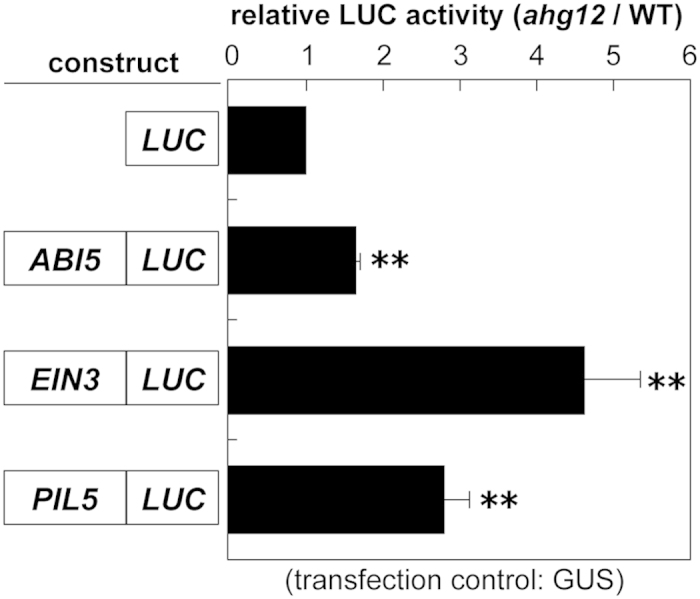
ABI5, EIN3, and PIL5 accumulate to a higher level in *ahg12*. Accumulation of LUC-fused proteins transiently produced in Arabidopsis protoplasts. The vectors for expressing LUC-fused proteins shown in the figure were co-transfected with the vector for expressing GUS. LUC activity in each sample was normalized to GUS activity. The data are means of three independent experiments. Error bars indicate standard deviation. (**P < 0.01; t-test).

**Figure 7:**
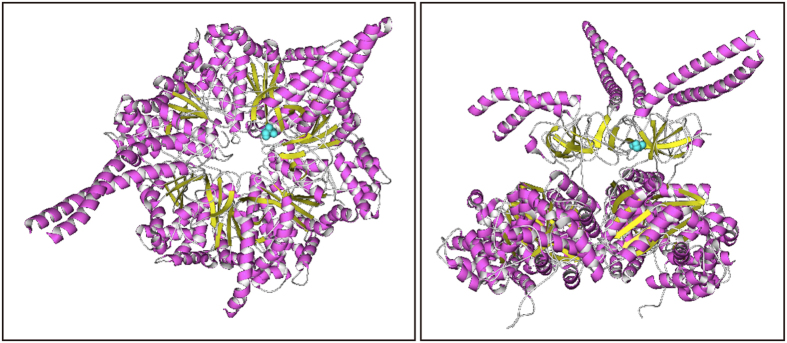
Predicted spatial localization of *ahg12.* Three-dimensional structure of the RP complex extracted from a published structural data of the 26S proteasome complex derived from budding yeast (PDB ID: 4CR2) (left panel: top view, right panel: side view). The amino acids corresponding to the *ahg12* mutation site of yeast RPT5 (Ser122) are represented with space-filling model (blue). The graphics were drawn with Molmil software.

Based on structural data for the 26S proteasome derived from *Saccharomyces cerevisiae*^27^, the *ahg12* mutation site, Ser112, was expected to face toward the pore through which substrate proteins are introduced into the CP (Fig. 7). This information implied that *ahg12* might affect the molecular mechanism for substrate uptake in the proteasome. ABA, ethylene, and light responses, which were changed in *ahg12*, are regulated by key regulators ABI5, EIN3, and PIL5, respectively^46^,^47^,^48^. The activities of these transcriptional regulators are modulated by protein degradation through the ubiquitin-proteasome system, responding to environmental stimuli^16^,^17^,^18^,^49^. Therefore, as next step, we investigated whether the *ahg12* mutation affects accumulation of these signaling components. For this purpose, the ORFs of these transcriptional factors were fused to *luciferase* (*LUC*) and the resulting recombinant proteins were transiently expressed in protoplasts derived from *ahg12* and wild-type plants. This assay allowed us to quantify the protein levels objectively^50^,^51^. The results of this assay demonstrated that the *ahg12* cells had higher levels of ABI5, EIN3, and PIL5 proteins than did the wild-type cells (Fig. 6). This result suggests that the *ahg12* mutation, which converts serine to the more bulky phenylalanine at the pore, decreases the degradation efficiency of the proteasome for some proteins, including ABI5, EIN3, and PIL5.

## Discussion

We isolated *ahg12* as an ABA-related mutant that showed a unique combination of phenotypes, with increased ABA and ethylene sensitivity and decreased light sensitivity at germination but also decreased seed dormancy. The *ahg12* mutation was found to create a novel amino acid substitution mutation in RPT5, which is a component of the RP of the 26S proteasome.

The *ahg12* mutation affected Ser112 of RPT5a. Although this amino acid residue is highly conserved among RPT5 proteins of eukaryotes, there has been no mutation around this residue reported previously. Structural studies of the *Mathanocoldococcus jannaschii* PAN complex, which is the archaeal counterpart of the eukaryotic proteasome complex, revealed that the residue corresponding to Ser112 of RPT5a is localized in loop L23 near the pore structure of the OB fold of the base subcomplex of RP^40^ (Fig. 7). It is likely that the conversion of an amino acid residue in this vicinity to the pore would affect the activity of the proteasome. Indeed, exchanging other residues in this loop of the PAN complex compromised PAN activity^41^. Since most of the proteasome structure of fission yeast overlaps with the PAN structure^42^, it is plausible that *ahg12,* wherein the Ser residue is converted to bulky Phe, affects RP activity in plants.

A question arises as to why the *ahg12* mutation affects restricted plant biological phenomena such as responses to ABA, ethylene, and light. It is possible that this mutation alters the substrate preference of the proteasome, and thereby decreases the degradation efficiencies of specific substrates including ABI5, EIN3, and PIL5. Functional asymmetry among RPT AAA-ATPases has been demonstrated in budding yeast^25^,^38^, in which a defect in each RPT somehow causes different effects on proteasome function. By analogy, *ahg12* in RPT5a might affect the degradation of a set of targets. This idea is consistent with the distinct effects of the *rpt2* and *rpt5* mutants of Arabidopsis^33^,^34^,^35^,^36^,^37^. The *ahg12* mutation might affect substrate preference due to a change in the pore structure of the RP as discussed above. It is also possible that *ahg12* slightly decreases the degradation efficiencies of all substrates. In this case, the phenotype related to the responses to ABA, ethylene, and light might be prominent in *ahg12* because these responses presumably require particularly drastic degradation of signaling components compared with other phenomena at germination. However, the fact that the ABA-hypersensitive phenotype was not observed in the null mutant of *RPT5a*, in which protein degradation capacity was probably lower than in *ahg12*, does not seem to support the latter possibility. In any case, isolation and characterization of *ahg12* demonstrated again that the responses to ABA, ethylene, light signals at germination are dependent on the protein degradation ability of the proteasome. Taken all together, our study on *ahg12* emphasizes the importance of protein degradation in the regulation of plant biological phenomena including germination, and confirms the necessity of the OB fold loop structure of the RP complex for proteasome activity.

The transient expression assays with LUC-fused proteins quantitatively demonstrated that the *ahg12* cells showed greater accumulation of signaling components that are involved in the physiological phenotypes of *ahg12* (Fig. 6). The difference in the accumulated ABI5 between *ahg12* and wild type was not particularly large in the leaf cell protoplasts, considering the clear ABA hypersensitive phenotype of *ahg12*. It is possible that the difference in ABI5 level is much larger in germinating seeds, where ABI5 naturally functions. It is likely that the stability of other substrates of the proteasome is also affected by *ahg12* and that these in turn presumably contribute to the phenotypes of this mutant. To examine what happens in the mutant in more detail, a comprehensive proteomic analysis is required.

Why are the genes encoding RPT proteins duplicated (e.g., *RPT5a* and *RPT5b*) in many plants? Why do various mutants of proteasome subunits show different phenotypes? These are major questions in the study of plant proteasomes. The amino acid sequence of RPT5b is quite similar to that of RPT5a, suggesting that these two RPT5 proteins could share functions to a large degree. In fact, a previous study reported that RPT5b can compensate for the absence of RPT5a in some cases^36^. In our study, the transgenic plants overexpressing *RPT5b* with an *ahg12*-like mutation did not show ABA hypersensitivity (Fig. S6), suggesting that there are some functional differences between RPT5a and RPT5b. It is possible that the slight differences in amino acid sequence are responsible for these distinct functions. Domain-swapping experiments between RPT5a and RPT5b might provide crucial data. In the future, we also plan to generate modified plants in which other RPT members have *ahg12*-like mutations and to evaluate the resulting phenotypes and proteasome substrate specificities. The identification of this dominant *RPT5a* mutant allele thus represents an important step toward addressing long-standing questions in proteasome biology.

## Methods

### Oligonucleotides

Oligonucleotides used in this study are listed in Table S1.

### Plant materials and growth conditions

*Arabidopsis thaliana* (L.) Heynh. ecotypes Columbia (Col) and Landsberg *erecta* (L*er*) were used. Plants were grown on MS plates (1× Murashige and Skoog salt mix, 2% sucrose, 2.5 mM MES (pH 5.8) and 0.8% agar) or on soil at 23 *°*C under 16 h light/8 h dark cycles. Seeds were first imbibed at 4 *°*C for 2 d before transfer to a growth chamber, unless otherwise noted. The *ahg12* mutant was isolated as described previously^39^ and the *aba2-1* mutation was removed by crossing with wild-type Columbia twice. The *RPT5a* T-DNA insertion line (*rpt5a-4*^37^, SALK_046321) was obtained from TAIR^52^ and given from Dr. Sakamoto (Tokyo University of Science). The *RPT5b* T-DNA insertion line (*rpt5b-3*^37^) was given from Dr. Yamaguchi (Hokkaido University).

### Mapping the *ahg12* locus

Rough mapping for the *ahg12* locus was described previously^39^. Genetic linkage analysis indicated that *ahg12* is located on chromosome 3. For fine mapping, genomic DNAs were extracted from approximately 800 F2 progeny between *ahg12* and L*er*, and genotyping was performed using PCR markers on chromosome 3. F3 seeds were obtained independently, and their ABA sensitivities were evaluated. Genotypes at the *ahg12* locus in the F2 individuals were determined based on the segregation ratio in F3 populations. Genes in the genomic region completely linked with the ABA-hypersensitive phenotype were analyzed by DNA sequencing.

### Generation of transgenic plants

The genomic *RPT5a*/*AHG12* gene with putative promoter and terminator regions was amplified from genomic DNA of the *ahg12* mutant by PCR with specific primers (Table S1). The DNA segment was cloned into a pGreenII 0129 binary vector^53^. The open reading frame of *RPT5b* was amplified from cDNA and then an *ahg12*-like mutation (Ser111 to Phe) was introduced into the *RPT5b* gene by PCR. Agrobacterium strain GV3101 was transformed with the plasmids and used for the transformation of Arabidopsis plants by the flower-dipping method^54^. Transgenic lines were screened by hygromycin tolerance.

### Immunoblot analysis

Total proteins were extracted from 2-week-old seedlings. Equal amounts of proteins were resolved by SDS-PAGE and transferred to PVDF membranes. Ubiquitinated proteins were detected by a polyclonal anti-ubiquitin antibody (BML-UG9510, Enzo Life Science) and HRP-conjugated anti-rabbit IgG antibody (W4011, Promega Corp.).

### Transient expression analysis of LUC-fused proteins

*ABI5*, *EIN3*, and *PIL5* cDNAs from the initiation codon to the last codon were amplified using specific primers (Table S1) and cloned into a plant expression vector to generate a fusion to the *LUC* gene under the control of the 35SCaMV promoter. These plasmids and a control plasmid containing a 35SCaMV promoter-GUS fusion were transfected into mesophyll protoplasts derived from the leaves of three-week-old *ahg12* and wild-type plants. The protoplasts were incubated at 22 *°*C under dark conditions for 24 h and harvested for LUC and GUS assays. The LUC and GUS activities were measured as described previously^55^.

## Additional Information

**How to cite this article**: Hayashi, S. and Hirayama, T. *ahg12* is a dominant proteasome mutant that affects multiple regulatory systems for germination of *Arabidopsis.* *Sci. Rep.* **6**, 25351; doi: 10.1038/srep25351 (2016).

## Supplementary Material

Supplementary Information
