# Supplementary material for: ahg12 is a dominant proteasome mutant that affects multiple regulatory systems for germination of Arabidopsis
Source: Sci Rep. 2016 May 3;6:25351. doi: 10.1038/srep25351 (PMC4853794; doi:10.1038/srep25351)
Supplement: Supplementary Information [file srep25351-s1.doc]

***ahg12* is a dominant proteasome mutant that affects multiple regulatory systems for germination of *Arabidopsis.***

**Shimpei Hayashi and Takashi Hirayama**

**SUPPLEMENTARY DATA**

**
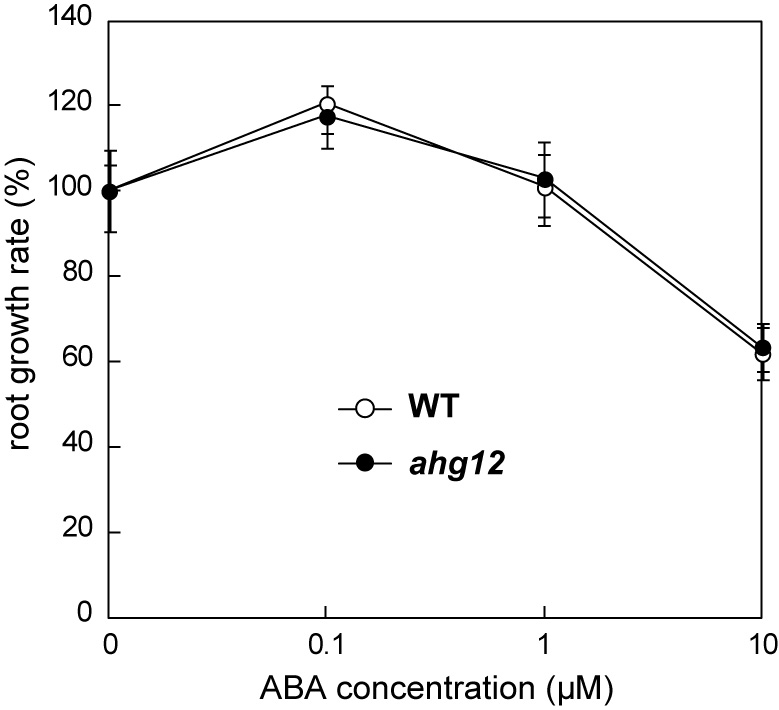
**

**Figure S1. ABA sensitivity of *ahg12* in root growth**

Root growth rate of *ahg12* seedlings grown on plates containing ABA. Root lengths of 4-day-old seedlings grown on normal plates were measured and then the seedlings were transferred to plates containing ABA at the indicated concentration. After 4 more days, root length was measured again and the growth rate calculated. The data are means of three independent experiments. Error bar indicates standard deviation.


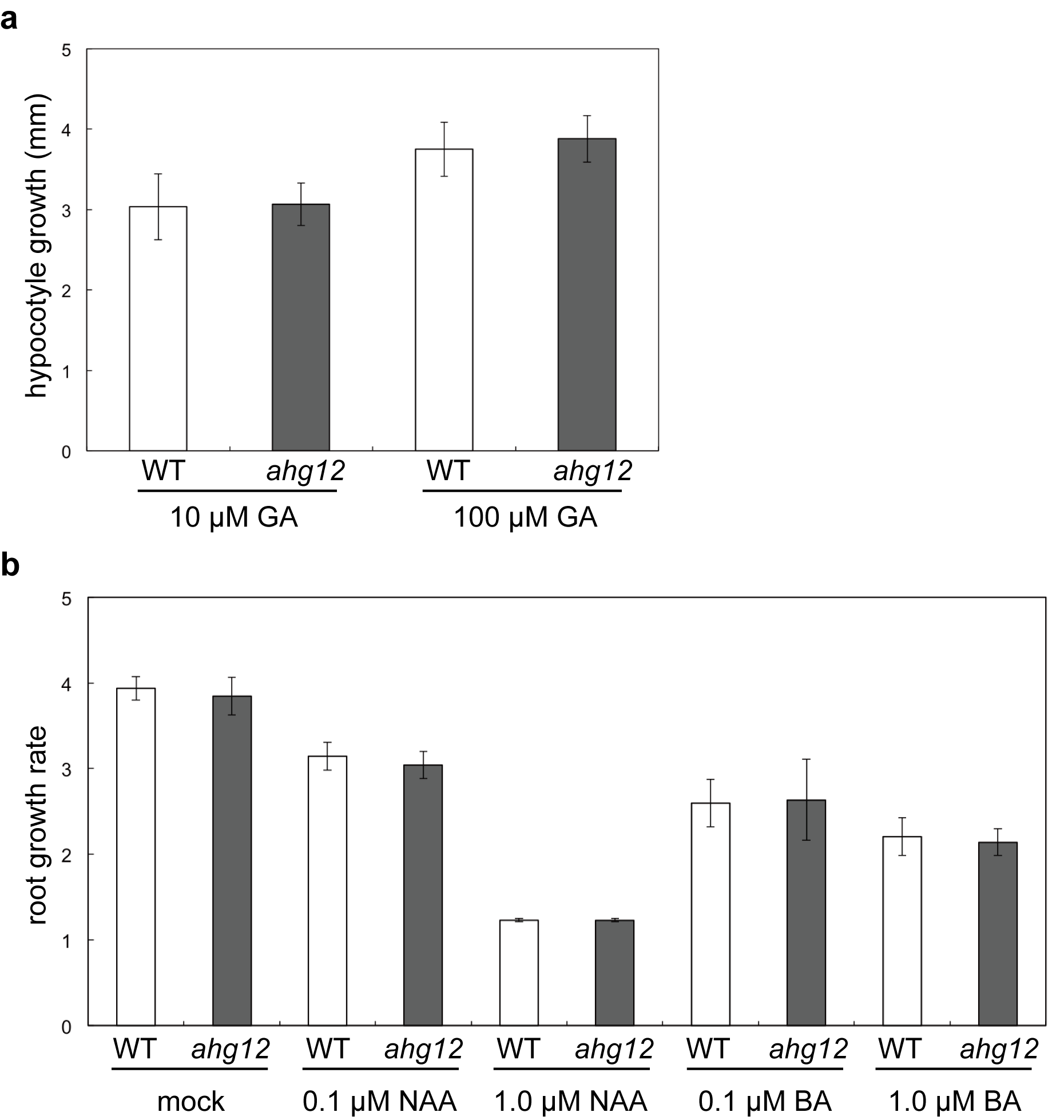


**Figure S2. Sensitivities of *ahg12* seedling to gibberellin, auxin and cytokinin**

(**a**) Hypocotyl length of *ahg12* seedlings grown on plates containing GA (gibberellic acid). Hypocotyl lengths of 8-day-old seedlings grown on plates containing GA at the indicated concentration were measured. (**b**) Root growth rate of *ahg12* seedlings grown on plates containing NAA (1-naphthaleneacetic acid) or BA (6-benzyladenine). Root lengths of 4-day-old seedlings grown on normal plates were measured and then the seedlings were transferred to plates containing NAA or BA at the indicated concentration. After 4 more days, root length was measured again and the growth rate calculated (root length after hormone treatment/root length before hormone treatment). Error bar indicates standard deviation.


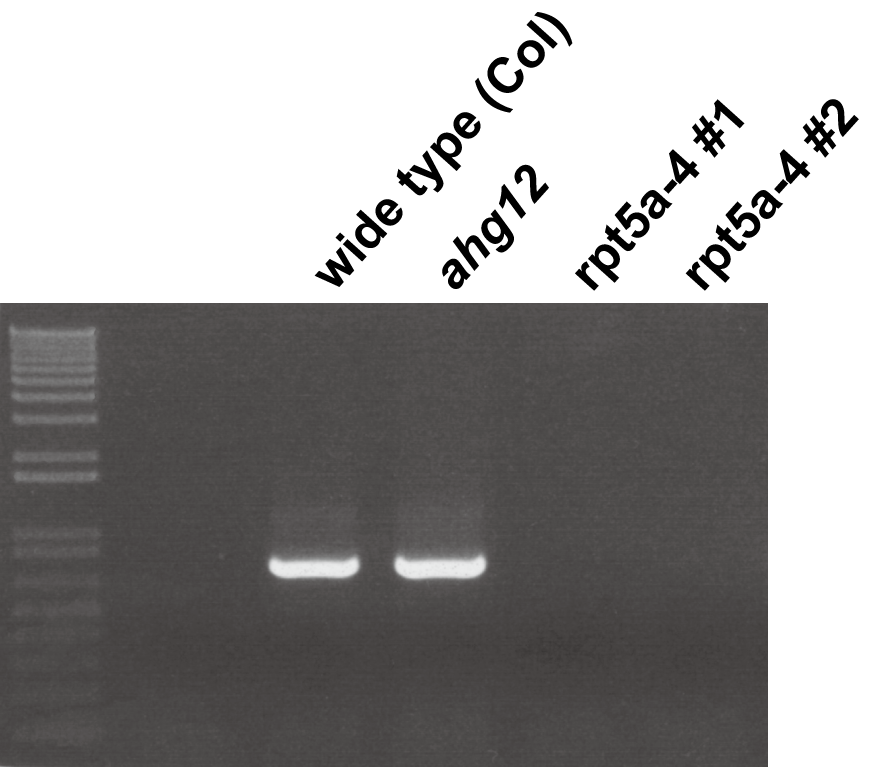


**Figure S3. No RPT5a transcripts in *rpt5a-4* (T-DNA insertion line).**

RT-PCR analysis for RPT5a transcripts was conducted with RPT5a F2 primer and RPT5a XhoStopR primer (see Table S1 online) using cDNA samples derived from wild type (Col), *ahg12*, *rpt5a-4* (two independent RNA samples).


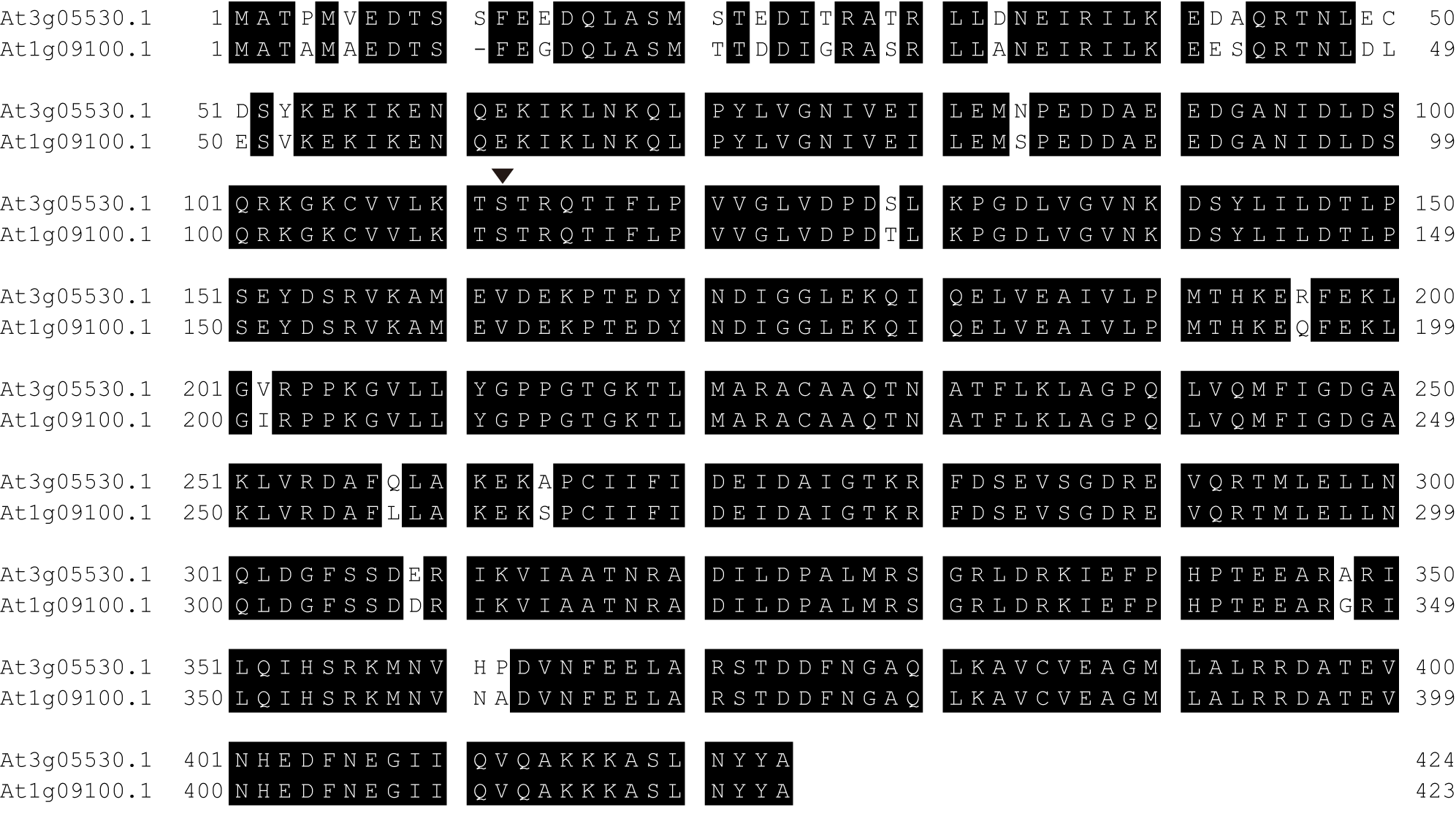


**Figure S4. Alignment of RPT5a and RPT5b**

Amino acid sequence alignment of At3g05530 (RPT5a) and At1g09100 (RPT5b). Completely conserved residues are indicated by black boxes. Triangle indicates the position of the *ahg12* mutation site (Ser112 to Phe).


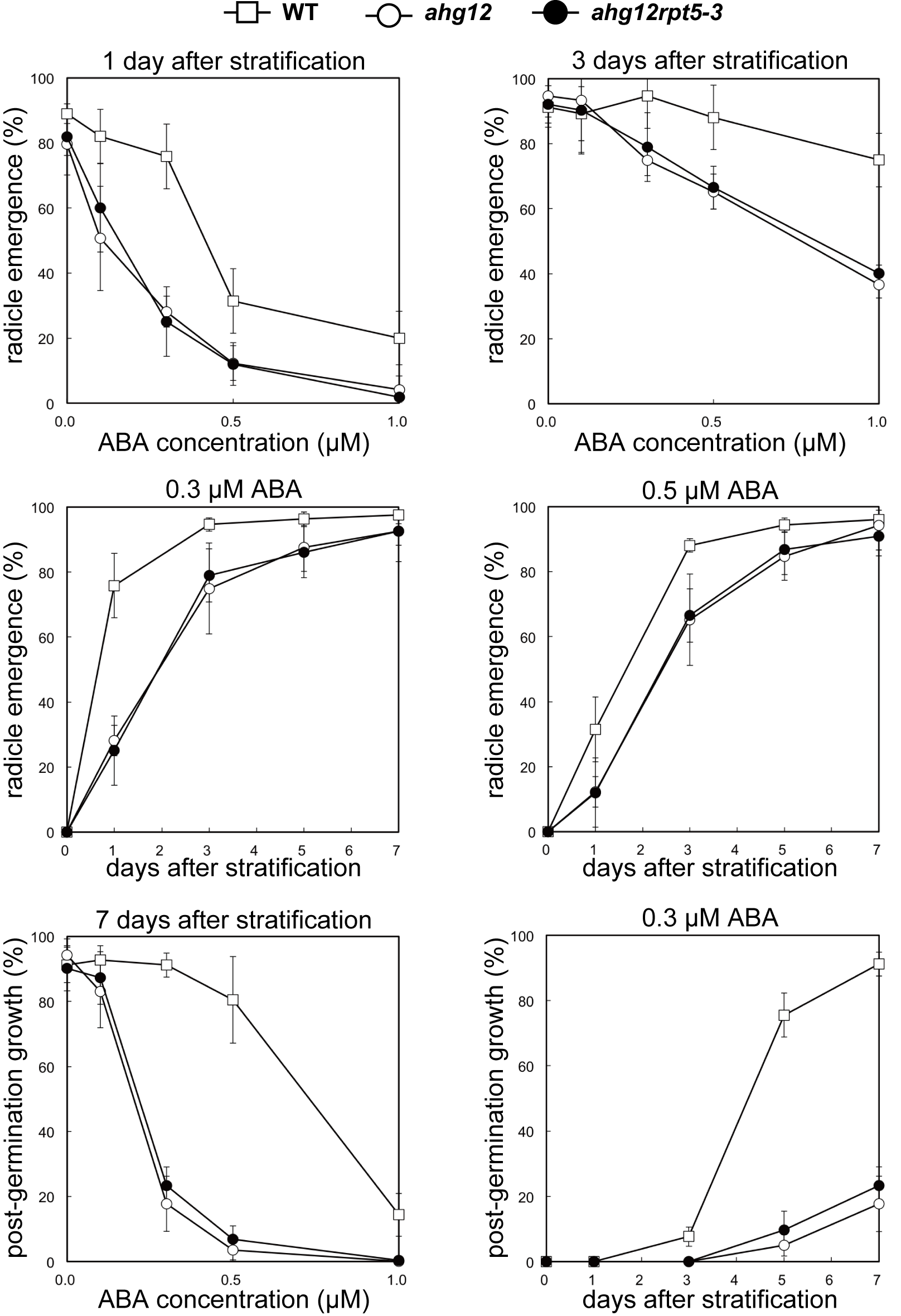


**Figure S5. Effect of *rpt5b-3* on the ABA hypersensitive phenotype of *ahg12*.**

Germination rate of wild type (Col), *ahg12* and *ahg12rpt5b-3* in the presence of ABA. Imbibed seeds (> 50) were stratified and then sown on plates containing ABA. Seeds that showed radicle emergence or post-germination growth (expansion of green cotyledons) were counted. The data are mean of three independent experiments. Error bars indicate standard deviation.


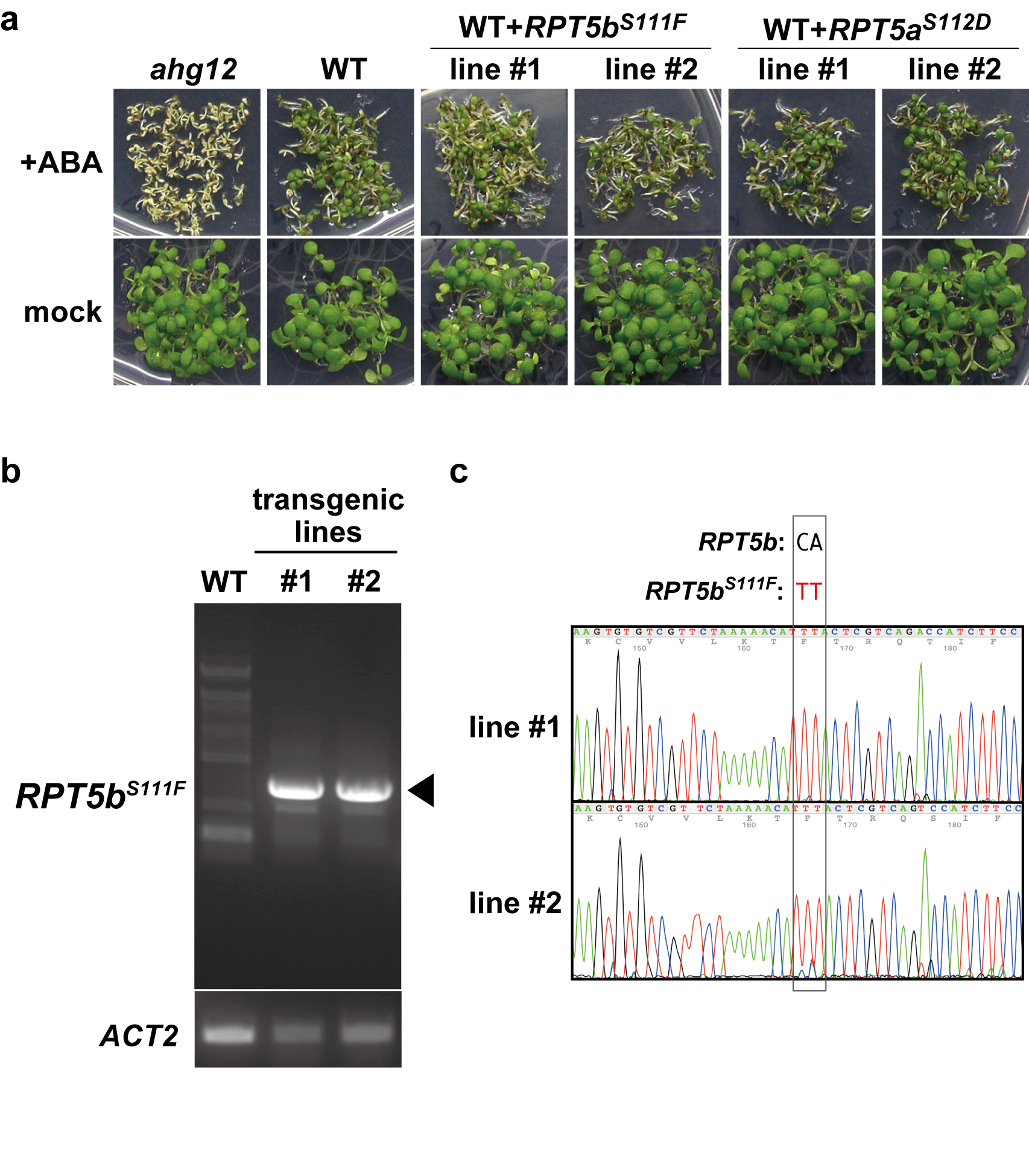


**Figure S6. ABA sensitivity of transgenic plants expressing *RPT5b* with an *ahg12*-like mutation.**

(**a**) Germinating seeds of transgenic plants that express *RPT5b* with an *ahg12*-like mutation or *RPT5a* with a S112D (phosphomimic) mutation. Imbibed and stratified seeds were grown on plates with or without ABA for 7 days. (**b**) RT-PCR analysis with RTP5b F2 primer and Nos-ter primer (see Table S1 online) using cDNA samples derived from the wild type or transgenic plants. Black triangle indicates the DNA fragment corresponding to the transgene. *ACT2* was used as a control. (**c**) Sequence analysis of the *RPT5b* transcripts. cDNA fragments containing the S111F mutation site were amplified with RPT5b F1 and F2 primers (see Table S1 online) using transgenic plant cDNA samples. The sequence chromatograms corresponding to the mutation site were shown. The wild type *RPT5b* sequence was hardly detected.
